# The Human Leukocyte Antigen G as an Immune Escape Mechanism and Novel Therapeutic Target in Urological Tumors

**DOI:** 10.3389/fimmu.2022.811200

**Published:** 2022-02-03

**Authors:** Simon Jasinski-Bergner, Markus Eckstein, Helge Taubert, Sven Wach, Christian Fiebig, Reiner Strick, Arndt Hartmann, Barbara Seliger

**Affiliations:** ^1^ Institute of Medical Immunology, Martin Luther University Halle-Wittenberg, Halle (Saale), Germany; ^2^ Institute of Pathology, Universitätsklinikum Erlangen, Erlangen, Germany; ^3^ Comprehensive Cancer Center Erlangen-Europäische Metropolregion Nürnberg (CCC ER-EMN), Erlangen, Germany; ^4^ Department of Urology and Pediatric Urology, University Hospital Erlangen, Friedrich Alexander University (FAU) Erlangen-Nürnberg, Erlangen, Germany; ^5^ Laboratory of Molecular Medicine, Department of Gynecology & Obstetrics, University Hospital Erlangen, Friedrich Alexander University (FAU), Erlangen-Nürnberg, Erlangen, Germany; ^6^ Main Department of GMP Cell and Gene Therapy, Fraunhofer Institute for Cell Therapy and Immunology, Leipzig, Germany

**Keywords:** HLA-G, renal cell carcinoma, epithelial bladder cancer, immune evasion, immunotherapy, immune cell infiltration

## Abstract

The non-classical human leukocyte antigen G (HLA-G) is a potent regulatory protein involved in the induction of immunological tolerance. This is based on the binding of membrane-bound as well as soluble HLA-G to inhibitory receptors expressed on various immune effector cells, in particular NK cells and T cells, leading to their attenuated functions. Despite its restricted expression on immune-privileged tissues under physiological conditions, HLA-G expression has been frequently detected in solid and hematopoietic malignancies including urological cancers, such as renal cell and urothelial bladder carcinoma and has been associated with progression of urological cancers and poor outcome of patients: HLA-G expression protects tumor cells from anti-tumor immunity upon interaction with its inhibitory receptors by modulating both the phenotype and function of immune cells leading to immune evasion. This review will discuss the expression, regulation, functional and clinical relevance of HLA-G expression in urological tumors as well as its use as a putative biomarker and/or potential therapeutic target for the treatment of renal cell carcinoma as well as urothelial bladder cancer.

## Introduction

During the last two decades it has been generally accepted that altered immune responses and immune evasion strategies are characteristic hallmarks of cancer. It has been demonstrated that the immune system within a tumor undergoes changes in its cellular composition, functionality and localization (1). While effector cells are often precluded from the invasive margin of tumors, immune suppressive effector cells are frequently found in this localization. Tumor extrinsic factors, like immune suppressive cells, soluble immune modulatory molecules, e.g. prostaglandin, arginase, metabolites or (anti-inflammatory) cytokines, will alter either the composition or the activity of tumor infiltrating lymphocytes (TILs) and promote tumor growth and metastasis (2). Further changes of the tumor microenvironment (TME) include an altered metabolism resulting in a low pH, hypoxia and chronic inflammation, which are predisposing factors and implicated in modulating the immune cell repertoire and contributing to immunological dysfunction (3, 4). Defects of immune sensing mediated by the expression of inhibitory immune checkpoint receptors (ICP-R), such as e.g. the program death-1 receptor (PD-1), the CTL-associated antigen-4 (CTLA-4), T cell immunoreceptor with immunoglobulin and ITIM domain (TIGIT), T cell immunoglobulin 3 (TIM-3), V-domain Ig suppressor of T cell activation (VISTA) and the lymphocyte activation gene (LAG-3) expressed on T and/or natural killer (NK) cells, represent so far known major immune escape mechanisms (5, 6). Thus, there is an urgent need for appropriate strategies in order to revert the immune suppressive TME to a more stimulatory milieu. In addition, tumor cells themselves are constantly developing strategies to escape immune surveillance, e.g. by altering the expression of classical and non-classical human leukocyte antigens (HLAs) and immune checkpoint ligands (ICP-L) and/or ICP-R (7). This will also lead to a reduced reactivity to innate and adaptive immune responses. Thus, a plethora of distinct features of tumor and immune cells limit the treatment efficacy and clinical outcome to cancer (immuno)therapies.

Novel cancer immunotherapies, such as immune checkpoint (ICP) inhibitors and adoptive cell therapy, have been developed during the last decade and demonstrated a therapeutic efficacy in hematologic tumors, but also in some solid cancers. However, the response rates to these therapies, in particular to solid tumors, still need to be improved and only a few patients achieve durable response rates due to intrinsic and/or acquired resistances mediated by immune evasion strategies of the tumor cells. This could be driven by both cellular and molecular suppressive networks within the TME, but also within the tumor, such as e.g. the loss of tumor antigens, downregulated expression of HLA class I molecules and antigen processing machinery (APM) and interferon (IFN) pathway components as well as upregulation of various immune checkpoint (ICP) molecules, like PD-L1, B7-H4, B7-H6, LAG-3, TIM-3, VISTA, HLA-E or HLA-G (8–10).

## General Features of HLA-G - Gene Structure, Expression, Regulation, Physiologic Function

HLA-G is a non-classical HLA class Ib molecule with a length of 4144 base pairs (bp) and is localized like the classical HLA class Ia molecules within the cluster of the major histocompatibility complex (MHC) on the short arm of chromosome 6 at region 6p21.3. However, it has distinct properties to HLA class Ia molecules (HLA-A, -B, -C) such as a highly restricted and tightly regulated expression, which is under physiological conditions limited to immune-privileged tissues/organs with confined local immune and inflammatory responses (11) preventing irreversible tissue damages. HLA-G expression was found on the cornea, but also on conjunctival and retinal pigment epithelial cells (12–14), insulin- and glucagon-positive cells within the endocrine islets of the pancreas (15), medullary thymic epithelial cells (16) and on extravillous trophoblasts of the placenta (17–19) protecting the fetus with its paternal antigens from maternal immune rejection (20–22). In these tissues, HLA-G contributes to immunological tolerance by acting as a ligand to inhibitory receptors expressed on several immune effector cells (23). Thus, HLA-G belongs to the few immunomodulatory proteins, whose main function lies in the mediation of a sufficient immunological tolerance even to foreign antigens. Although HLA-G is not physiologically expressed in most adult tissues, neoexpression/(re)expression of HLA-G is a frequently observed phenomenon in different cancers thereby inducing an immunological tolerance and suppression of immune surveillance (18, 19, 24). Due to alternative splicing, the HLA-G protein can exist as membrane-bound isoforms and soluble forms (25–27), which bind to inhibitory receptors of immune effector cells thereby inhibiting immune responses (28, 29): (i) leukocyte immunoglobulin-like receptor, subfamily B, member 1/LILRB1 (synonym: Ig-like transcript 2/ILT2; CD85J), (ii) leukocyte immunoglobulin-like receptor, subfamily B, member 2*/*LILRB2 (synonym: Ig-like transcript 4/ILT4; CD85D), (iii) killer cell immunoglobulin-like receptor, two domains long cytoplasmic tail, 4/KIR2DL4 (CD158D), (iv) killer cell lectin-like receptor, subfamily c, member 1/KLRC1 (synonym: natural killer cell lectin; NKG2A) and (v) natural killer cell receptor By55 (CD160). LILRB1 is expressed on monocytes, dendritic cells (DCs), B cells, NK cells and T cells; LILRB2 on cells of myeloid origin, KIR2DL4 on NK cells, KLRC1 on approximately 50% of NK cells and on a subset of CD8^+^ T cells and CD160 is expressed on NK cells. Furthermore, HLA-G can be expressed and secreted from non-cancer cells, such as human mesenchymal stem cells (hMSCs). These hMSCs can inhibit both NK cell cytotoxicity and T lymphocyte alloproliferation (30). In addition, the HLA-G receptor LILRB2 (ILT4) has been shown to be expressed on hematopoietic stem cells supporting their stemness through binding to angiopoietin-like proteins (29).

The expression of LILRB1/ILT2 and LILRB2/ILT4 on different immune effector cells, endothelial cells and tumor cells and the effects of the interaction with HLA-G have been recently summarized by Carosella and co-authors (31), while the different affinities of the HLA-G receptors to HLA-G as ligand and the exact binding positions have been reviewed elsewhere (26).

Another different feature of HLA-G compared to the HLA class Ia molecules is the low number of HLA-G alleles. To date (October 2021) the IPD and IMGT/HLA database (32) enlists 88 different HLA-G alleles compared to the several thousands of different HLA-A/B/C alleles. Furthermore, alternative splicing is commonly found within the human *HLA-G* gene leading to at least seven different HLA-G protein encoding splice variants (HLA-G1-HLA-G7) reviewed by Hviid, 2006 (33). Next to the primary transcript of HLA-G (HLA-G1), containing the α-1, -2 and -3 domains as well as a transmembrane (TM) domain resulting in a membrane-bound protein, several other membrane-bound and soluble HLA-G isoforms have been described (34–37) (**Table 1**).

**Table 1:** Overview of all described HLA-G isoforms.

HLA-G isoforms	Alternative Splicing	Effect	Reference
HLA-G1	wild type	membrane-bound	*(* 34 *–* 36 *)*
		heavy chain (HC) with α-1, α-2, α-3 domain and transmembrane domain (TM)	
HLA-G2	no exon 3	membrane-bound	(34)
		lack of α-2	
HLA-G3	no exon 3 and 4	membrane-bound	(34)
		lack of α-2 and α-3	
HLA-G4	no exon 4	lack of α-3	(34)
HLA-G5	includes intron 4	no TM domain; soluble;	(35)
	translation stops after exon 4	HC with α-1, α-2, α-3	
HLA-G6	includes intron 4 translation stops after exon 4	no TM domain; soluble; lack of α-2	(35)
	no exon 3		
HLA-G7	includes intron 2	no TM domain; soluble;	(35)
	translation stops after exon 1	lack of α-2 and -3	
HLA-G1L	5 additional N-terminal amino-acids (MKTPR)	membrane-bound	(36)

TM, transmembrane domain; intron 5 was previously known as intron 4 according to the IMGT/HLA nomenclature.

While the isoforms HLA-G1 to HLA-G4 encode for membrane-bound proteins, the isoforms HLA-G5 to HLA-G6 contain the unspliced intron 4, HLA-G7 the unspliced intron 2 resulting in an early stop codon and leading to the loss of the transmembrane domain of HLA-G. As a consequence, the proteins HLA-G5 to HLA-G7 are soluble and secreted thereby contributing to changes in the local and peripheral microenvironment (38). The most abundant HLA-G proteins are HLA-G1 and HLA-G5, which both consist of a heavy chain with three domains (α1, α2, α3). In both cases the heavy chains are associated with the β_2_-microglobulin (β_2_-m) and these complexes can even present a limited and cell type-specific peptide repertoire towards CD8^+^ cytotoxic T lymphocytes (CTL) (39), which is not a prerequisite for their inhibitory functions towards immune effector cells.

In contrast, the other HLA-G isoforms are not bound to β_2_-m lacking one or two α domains, e.g. HLA-G2 (has α1 and α3), HLA-G3 (α1), HLA-G4 (α1 and α2), HLA-G6 (α1 and α3), and HLA-G7 (α1) (33). Not only the alternative splicing, but also the proteolytical shedding of membrane-bound HLA-G proteins mediated by the matrix metalloproteinase (MMP) 2 leads to the generation of sHLA-G protein isoforms (40, 41). It is noteworthy that in addition to the functional role of membrane-bound and soluble HLA-G, the knowledge of the splicing pattern must be considered for the choice of respective antibodies for quantification and/or identification of the cellular localization. Recently, a number of antibodies directed against the different HLA-G isoforms have become available for appropriate protein detection and quantification as summarized by Krijgsman and co-authors (42). Their use in combination with antibodies staining HLA-G receptors demonstrated a heterogeneous expression leading to a fine-tuned network regulating the HLA-G-mediated immune interaction (42).

**Figure 1:**
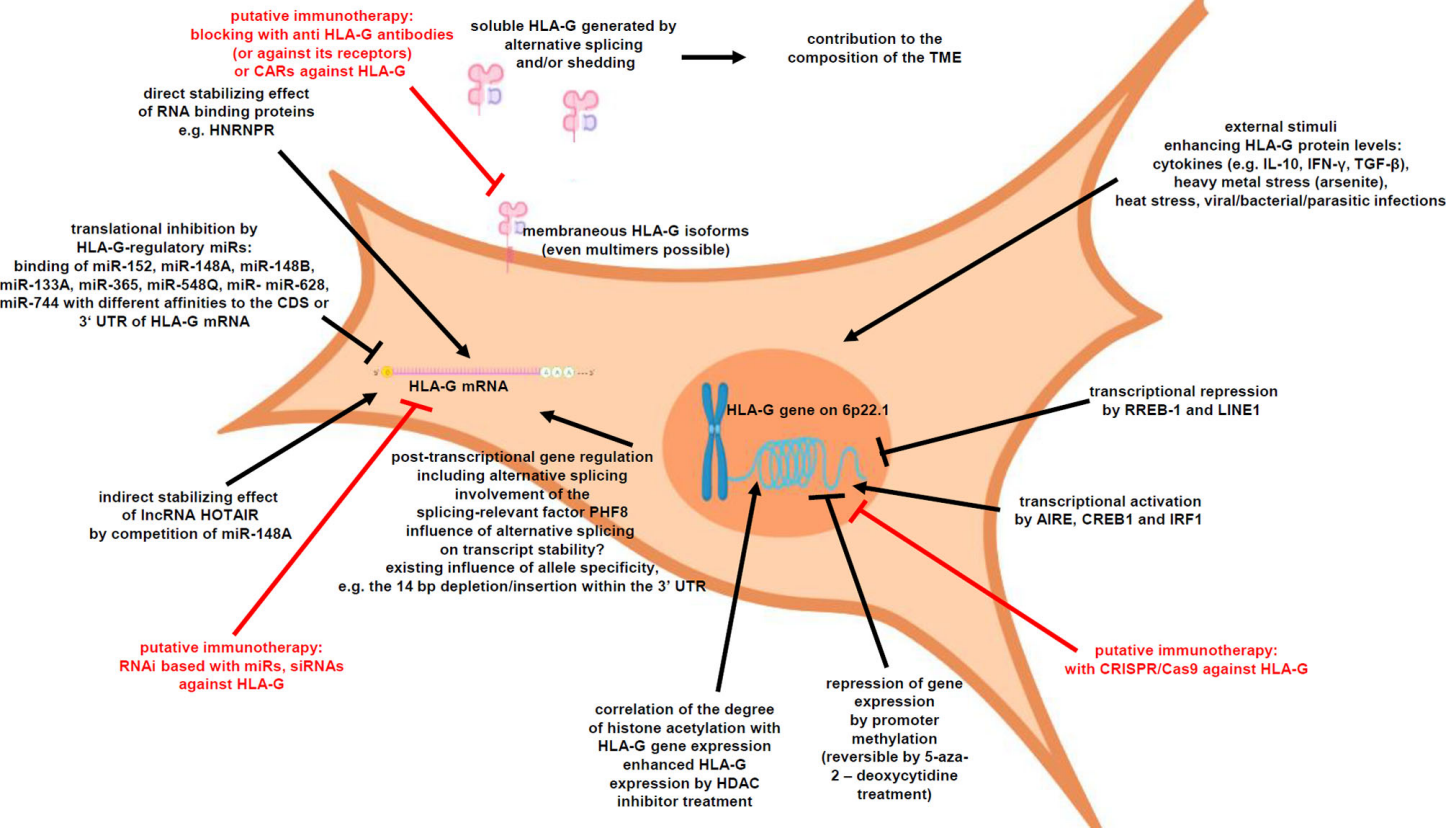
Underlying mechanisms of altered HLA-G expression.

HLA-G expression is tightly regulated at different levels (**Figure 1**), such as transcriptional, posttranscriptional as well as epigenetic control. Indeed, a HLA-G promoter methylation can be frequently found in HLA-G-negative cells, which could be reversed by demethylating agents thereby inducing HLA-G transcription (43, 44). DNA methylation of its promoter is based on the covalent binding of a methyl (-CH_3_) group to a cytosine residue in CpG dinucleotides enriched CpG islands (45). The degree of CpG methylation at the HLA-G promoter is associated with HLA-G expression (46). Chromatin immunoprecipitation demonstrated a differential histone acetylation status of HLA-G^+^-expressing vs. HLA-G-non-expressing cells. Treatment with histone deacetylation inhibitors (HDACi), like sodium butyrate or trichostatin A (TSA), induced histone hyperacetylation, which was associated with a reversion of HLA-G repression (47). In addition to epigenetic mechanisms, HLA-G expression is transcriptionally regulated. In this context, several HLA-G relevant transcription factors have been reported. In the HLA-G expressing medullary thymic epithelial cells, the transcription factor AIRE has been identified to increase HLA-G transcription (48), but it has to be mentioned that the AIRE expression dramatically decreases by age-related thymic involution (49, 50), which could affect indirectly the HLA-G expression. Recently, the cAMP-responsive element binding protein (CREB)1 and IRF1 have been demonstrated to bind HLA-G promoter sequences leading to an increased HLA-G transcription (51–53). In contrast, RREB-1 and LINE1 repressed HLA-G gene transcription (54, 55). Furthermore, several stress stimuli have been reported to increase HLA-G transcription, including heat stress, heavy metal stress, viral/bacterial/parasitic infections [as reviewed by L. Amiot (56)] and several cytokines, such as IL-10, IL-6 and IFN-γ (46, 57, 58).

Next to the transcriptional regulation, HLA-G expression is posttranscriptionally controlled by microRNAs (miRNAs), RNA-binding proteins (RBPs) and long non-coding RNAs (lncRNAs) (59–61). A number of miRNAs have been reported to directly bind to the HLA-G mRNA and interfere with the HLA-G translation leading to HLA-G mRNA decay: miR-152, miR-148A, miR-148B, miR-133A, miR-365, miR-548q, miR-628, and miR-744 (57, 62–66). These miRNAs usually bind either to the 3’-untranslated region (UTR) or in the case of miR-744 to the coding sequence (CDS) of HLA-G. An allele specificity is the 14 nt deletion/insertion within the 3’-UTR of the HLA-G mRNA in regard to the binding of regulatory factors including miRNAs (67). Recently, a distinctive site only present in the 3’ UTR of HLA-G was identified, which binds RBPs and miRNAs (68). In addition, the RBP HNRNPR has been identified to regulate HLA-G, but also classical HLA class I antigens (60), while the lncRNA HOTAIR modulates HLA-G expression by competitively binding the HLA-G regulatory miR-148A and miR-152 (66, 69).

## HLA-G Expression in Solid and Hematopoietic Tumors and Its Clinical Relevance

It has been recently demonstrated that HLA-G is crucial for tumor immune evasion and is also associated with malignant transformation (29). A pathophysiological expression of HLA-G was frequently detected on the cell surface of solid and hematopoietic malignancies (70). These include breast cancer, non-small cell lung cancer, esophageal squamous cell carcinoma, gastric cancer, colorectal cancer, hepatocellular carcinoma, oral squamous cell carcinoma, cervical cancer, ovarian cancer, bladder cancer, pancreatic cancer, glioma, renal cell carcinoma, and thyroid cancer as well as leukemia and lymphoma (**Table 2**). In contrast, HLA-G expression in adjacent healthy tissues has not been detected. Next to membranous HLA-G expression, sHLA-G isoforms were often elevated in plasma or serum samples of tumor patients (85, 86). In addition, HLA-G has been detected in extracellular vesicles (EV) in the supernatant of tumor cells including melanoma cells and might play a role in cancer immune escape by inhibiting immune cells in the TME even at distal sites (87).

**Table 2:** Summary of pathological HLA-G neoexpression in solid and hematopoietic malignancies.

Tumor Entity	Cell Lines/tumor Samples/Plasma Samples; Number of Samples	Method/Applied Antibody	Frequency of HLA-G Expression	Correlation of the HLA-G with Clinical Parameters	References
breast cancer	235 primary breast cancer lesions; 44 plasma samples of breast cancer patients and 48 plasma samples of healthy controls	IHC (mAb HGY), ELISA	66% HLA-G positive breast cancers; sHLA-G: 0.74 μg/mL in stage I patients, sHLA-G: 0.78 μg/mL in stage II patients; sHLA-G: 0.43 μg/mL in healthy donors	statistically significant correlation tumor size (p = 0.0001), nodal status (p = 0.012), disease stage (p = 0.0001), HLA-G positive patients with lower survival rate (p < 0.028); elevated sHLA-G levels in plasma of breast cancer patients (p < 0.001)	(71)
breast cancer	677 early breast cancer lesions	IHC (mAb 4H84)	60% HLA-G positive breast cancers	predictor for breast cancer patients	(72)
cervical cancer	22 normal cervical tissues, 14 cervical intraepithelial neoplasia patients,	IHC (mAb 4H84)	0% in normal cervical tissues, 35.7% in cervical intraepithelial neoplasia, 62.8% in squamous cell cervical cancer patients	association with disease progression	(73)
	129 patients with squamous cell cervical cancer				

cervical cancer	22 normal cervical tissues, 119 primary cervical lesions;	IHC (mAb 4H84),	0% in normal cervical tissues,	significant correlation (p < 0.05) to size of the main lesion, parametrical invasion and lymph node metastasis	(74)
	172 plasma samples of patients with cervical cancer and 20 plasma samples of healthy controls	ELISA (MEM-G/9)	45% in primary cervical lesions;		
			statistically significant higher sHLA-G levels in plasma of cervical patients (median 191.4 U/ml) versus plasma of healthy controls (median 45.18 U/ml, p < 0.001)		
colorectal cancer (CRC)	457 primary colorectal cancer (CRC) (colon = 232, rectal = 225 lesions)	IHC (mAb 4H84)	70.7% HLA-G positive CRC specimen	significant association with worse prognosis (p = 0.042)	(75)
colorectal cancer	144 plasma samples of CRC patients,	ELISA (MEM-G/9)	statistically significant (p < 0.01) increased sHLA-G plasma levels in CRC patients (median 124.3 U/ml) than in healthy controls (median 25 U/ml)	no correlation	(76)
	60 plasma samples of healthy controls				
gastric cancer	94 unselected patients with gastric adenocarcinoma	IHC (mAb, 4H84)	25.5% HLA-G positive gastric adenocarcinoma specimen	significant association with (p < 0.0001), worse survival	(77)
	(stage I to III)				
glioblastoma	108 glioblastoma specimen	IHC (mAb, MEM-G/02)	60.2% HLA-G positive glioblastoma specimen	negative effects on the survival rate	(78)
hepatocellular carcinoma	74 primary hepatocellular carcinoma specimen	IHC (mAb, 4H84)	31% HLA-G positive hepatocellular carcinoma specimen	no correlation	(79)
ovarian cancer	169 primary ovarian carcinoma lesions with type II, high grade serous and undifferentiated	IHC (mAb 4H84)	47.9% HLA-G positive primary ovarian cancer specimen	significant correlation with a favorable prognosis	(80)
				(p = 0.038)	
ovarian cancer	33 primary ovarian carcinoma lesions,	IHC (mAb 4H84)	66.7% HLA-G positive primary ovarian cancer specimen,	protection from NK cell lysis	(81)
	13 normal ovarian tissues		0% of the normal ovarian tissues	(*in vitro*)	
pancreatic carcinoma	42 primary pancreatic carcinoma specimen	IHC (mAb, 4H84)	66% HLA-G positive pancreatic carcinoma specimen	significant correlation with grade (p=0.007), stage (p=0.038) and poor prognosis	(79)
testicular cancer	34 primary testicular cancer patients	IHC (mAb 4H84)	20.6% HLA-G positive samples	no correlation	(82)
chronic	68 chronic myeloid leukemia	ELISA (sHLA-G1, HLA-G5)	association of sHLA-G with HLA-G alleles	reduced event free survival	(83)
myeloid					
leukemia					
chronic lymphocytic leukemia	45 chronic lymphocytic leukemia	flow cytometry (MEM-G/9)	1 – 12% positive	independent prognostic factor	(84)

Since HLA-G is frequently associated with an advanced tumor stage and a poor prognosis of patients, a diagnostic and prognostic potential has been suggested for HLA-G in different cancer types.

## Frequency of HLA-G Expression in Renal Cell Carcinoma

### Characteristic Features of RCC

Renal cell carcinoma (RCC) is the most common kidney tumor with an incidence of 2% of tumors in the Western world (88). Based on the histology several RCC subtypes were classified, with clear cell (cc)RCC as major subtype with an incidence of 75 % of all RCCs, followed by papillary and chromophobe RCC with an incidence of 10 % and 5%, respectively and other subtypes accounting for less than 1 % (89). Hypertension, smoking, diabetes mellitus, chronic kidney diseases, kidney cysts, kidney transplantation are known risk factors for this disease with a clear gender imbalance (1 female patient to 1.8 male patients) (90). In addition, genetic predispositions, like mutations in tumor suppressor genes, e.g. the von Hippel Lindau (VHL) and PTEN gene, have been reported (88, 91).

During the last decades, the therapeutic options of RCC improved starting from unspecific cytokine treatment using high doses of IL-2 for lymphocyte activated killer cell generation (92), followed by application of receptor tyrosine kinase inhibitors, like sorafenib, sunitinib and axitinib, or the mTOR inhibitors temsirolimus and everolismus. More recently, therapeutic mAbs directed against ICP axes, namely the PD1 and PD-L1 axis (e.g. nivolumab, durvalumab) as well as the CTLA-4 and B7-1/B7-2 axis (e.g. ipilimumab) were developed and introduced into the treatment regimens. Actual research focuses on the identification of novel ICPs as well as the development of mAbs directed against LAG-3, TIM-3, TIGIT and other ICP-R and/or ICP-L for mono- or combination therapy (93).

It is noteworthy that HLA-G neoexpression and/or LILRB2/ILT4 expression in ccRCCs has been recently linked to aberrant expression of the vascular endothelial factor (VEGF)A and VEGFC (94). Thus, receptor tyrosine kinase inhibitors, which block predominantly the neoangiogenesis by inhibiting the tyrosine kinase domain of the VEGF receptors, might be used in combination with anti-HLA-G therapies.

### Frequency of HLA-G Expression in RCC

While HLA-G expression could not be detected in healthy renal tissue, the pathophysiological HLA-G expression in RCC lesions ranged between 30 and 60% and was found at the cell surface as well as in the cytoplasm. Furthermore, the HLA-G plasma levels were statistically significant increase in RCC patients (**Table 3**). In ccRCC, a high frequency of HLA-G mRNA and protein expression has been described, which is age and sex independent, while in other RCC subtypes HLA-G expression was not detected. **Table 3** summarizes the frequency of HLA-G expression in RCCs, which was determined by different methods including immunohistochemistry (IHC), Western blot (WB) analysis and/or PCR for HLA-G detection as well as its clinical relevance.

**Table 3:** Frequency of HLA-G expression and its clinical relevance in RCCs.

Cell Lines/tumor Samples/plasma Samples; Number of Samples	Method/Applied Antibody	Frequency of HLA-G Expression	Clinical Relevance	Study
18 primary RCC lesions	IHC (4H84)	primary RCC: 61.1%	none	(95)
with adjacent renal tissue				
37 primary RCC lesions with adjacent renal tissue;	WB (mAbs MEM-G/9 and MEM-G/1)	primary RCC lesions: 27% RCC cell	none	(96)
24 RCC cell lines and 8 autologous normal kidney cells	qPCR	lines: 12.5% mRNA positive, RCC cell lines: 8.3% protein positive		
14 RCC cell lines	WB (mAb	mRNA positive: 57%	n.a.	(43)
	4H84), qPCR	protein positive: 43%		
109 primary RCC lesions,	IHC/WB (mAb 4H84);	primary RCC lesions: 47.7%	none	(97)
34 adjacent tumor negative renal tissue,	ELISA (MEM-G/9)	ccRCC: 49.5%		
16 plasma samples of RCC patients		chromophobe: 50% (n: 2/4)		
		collecting duct RCC: 50% (n: 3/6) RCC		
		sHLA-G in RCC patients:		
		39.5 U/ml		
		normal controls: 19.2 U/ml (P = 0.002)		
453 primary RCC lesions	IHC (mAb 4H84)	RCC samples: 49.9% membranous: 38.1% cytoplasmic expression	higher frequency of stronger cytoplasmic HLA-G staining in grade 3 tumors than lower grade tumors (p = 0.014)	(57)
33 plasma samples of RCC patients and healthy control group	ELISA	sHLA-G levels in RCC (46.6 U/ml) than in HC (18.3 U/ml); (p = 0.41)	correlation of higher sHLA-G levels with advanced tumor stage and progression	Rodrigo et al., 2016 (DOI: 10.1200/JCO.2016.34.15_suppl.e16066 Journal of Clinical Oncology 34, no. 15_suppl)
	(MEM-G/9)			

n.a., not analyzed.

Omitting early reports applying only RCC cell lines, studies analyzing RCC patient cohorts could demonstrate a statistically significant correlation between HLA-G expression and higher tumor grading and staging in RCC patients using IHC and ELISA. These data suggest that HLA-G might be an interesting prognostic marker for RCC.

Furthermore, a link between the pathophysiologic HLA-G expression and the frequency and composition of the immune cell infiltration was reported (57). The HLA-G expression in RCC appears to be also associated with an altered immune cell repertoire in the TME. These TILs did not express CD25 and CD69 activation markers within the HLA-G positive group confirming the hypothesis that HLA-G expression might contribute to the immune evasion of the tumor cells by inhibition of immune effector cells. Interestingly, the HLA-G receptors LILRB2/ILT4 were detected in stromal macrophages, plasma cells and infiltrating lymphocytes of RCC samples suggesting the presence of an immune-tolerant microenvironment.

Analysis of mRNA and protein levels revealed a discordant HLA-G mRNA and protein expression pattern frequently occurred suggesting a post-transcriptional regulation of HLA-G in this tumor entity (70, 98). Furthermore, a loss of HLA-G mRNA and cell surface expression of ccRCC cells was observed during cell culture, which might be explained by the absence of TFs modulated by the hypoxic microenvironment, the lack of cytokines, such as IFN-γ, IFN-α and IL-10, or an increasing promotor methylation (96).

## HLA-G Expression in Bladder Cancer

Urothelial bladder cancer (BC) is the 10^th^ most common tumor worldwide with a high incidence in the Western world. It is more prevalent in male than female (99). The initiation and progression of BC is a multifactorial process and comprises of immune surveillance, immune balance and immune escape (100). There exists accumulated evidence that BCs evade immune surveillance by modulating immune suppressive networks in the TME and upregulating co-inhibitory molecules like PD-L1 and HLA-G (101, 102). A heterogeneous pathophysiological neoexpression of HLA-G has been demonstrated in various stages of urothelial BC ranging from a frequency between 16.7 to 68 %, whereas adjacent normal urothelium lacks HLA-G expression (103, 104). In contrast, sHLA-G levels in serum of bladder cancer patients and healthy controls did not differ. Furthermore, higher levels of HLA-G transcripts than HLA-G protein were found in bladder cancer suggesting a posttranscriptional control comparable to that of RCC lesions. However, HLA-G-regulating mRNAs have not been yet investigated in bladder cancer (104).

In addition, repeated applications of Bacillus Calmette Guerin (BCG) can induce HLA-G neoexpression extrinsically leading to acquired resistance to further BCG-based instillation therapy (105). Few studies also demonstrated that patients with non-muscle-invasive urothelial carcinoma have an increased prevalence of peripheral blood T cells that are susceptible to HLA-G-mediated immunosuppression through co-expression of ILT2 and ILT4 (106). High peripheral prevalence of T helper cells and CTL expressing ILT2 is also associated with an increased risk of recurrence of non-muscle invasive urothelial carcinoma (107). However, no other published data currently exist on the significance of HLA-G in urothelial BC. Due to the specific immunological function of HLA-G and the overall sobering results of conventional ICP-oriented immunotherapies, further research on the role and potential therapeutic target ability of HLA-G in local and advanced stages of urothelial BC is required.

To best of our knowledge meaningful studies with sufficient BC or RCC patient cohort sizes in regard to elevated HLA-G protein levels in urine samples and its suitability as a potential prognostic marker has not yet been performed.

## Intratumoral Heterogeneity of HLA-G Expression in RCC and BC

A strong intratumoral heterogeneity exists in RCC for the expression of ICPs, such as PD-L1, B7-H3, PD-L2 and HLA-G, in primary RCC lesions with a highly variable HLA-G expression either between patients’ tumor samples or at different areas within a tumor tissue (36, 108). Tronik-Le Roux and co-authors showed that ccRCC tumors were strongly, diffusely positive or negative using an antibody (ab) directed against the HLA-G alpha-1 domain (36). However, using an antibody specific for amino acids of intron 4 recognizing HLA-G without a transmembrane domain, IHC staining results were highly variable ranging from weak, negative to strong staining (36). Due to the assumption that the HLA-G α-1 domain is present in all HLA-G isoforms, the results of HLA-G expression would have been negative for patient tumors according to the α-1 specific antibody, but were strongly positive for the intron 4 (=5) specific antibody (36). Another study also showed intratumoral heterogeneity in ccRCC patients (108) with very heterogeneous staining pattern for HLA-G ranging between 37 – 70% of HLA-G throughout one tumor using IHC as well as for ILT4, which preferentially binds HLA-G. In contrast, a different RCC tumor lack HLA-G expression, but exhibit heterogeneous staining of ILT4. Interestingly, the HLA-G staining was inversely correlated to the PD-L1 staining (108). A larger study with 109 mixed RCC lesions demonstrated HLA-G expression in different RCC subtypes except for papillary RCC and control tissues. Soluble HLA-G in plasma of RCC patients showed higher expression compared to controls (97).

Immunohistochemical analysis of 75 primary bladder transitional cell carcinoma (TCC) lesions demonstrated a HLA-G expression in 51 of 75 tumors while the normal bladder lacks HLA-G expression. However, the expression varied from negative to 100% positive (103). A similar intratumoral heterogeneity of HLA-G expression was also shown in colorectal cancer (CRC) using different HLA-G specific antibodies (109).

## Clinical Relevance of HLA-G Expression in RCC and Bladder Cancer

Due to the frequent pathophysiological HLA-G expression in solid and hematopoietic tumor entities a clinical relevance has been suggested. In **Table 2**, selected studies investigating the HLA-G expression in solid tumor entities with meaningful patient cohort sizes are summarized and correlations to clinical parameters are highlighted.

As shown in **Table 2**, a correlation of HLA-G expression with disease progression, tumor size and in some cases also with prognosis of RCC exists. It is noteworthy that the expression intensity of the HLA-G protein had an impact on clinical relevant parameters, such as tumor staging/grading, disease progression and patients’ survival. In addition to alternative splicing of HLA-G transcripts, deletions, insertions and nucleotide polymorphisms of the *HLA-G* gene have been described, which are important parameters for cancer and clinical correlations (110). In a study with 56 metastatic RCC, the 14 bp insertion/deletion polymorphism in the 3’ UTR was analyzed (111). A trend towards better patients outcome was demonstrated in the presence of the homozygosity for the 14 bp deletion allele, while a better patients’ survival for RCC with heterozygotic T/C vs. homozygotic T/T nucleotide polymorphism at p3003 was detected. Other allelic groups of HLA-G (G*0104 and G*0103) were found to be associated with the susceptibility to urinary bladder papillary transitional carcinoma (112). Next to the HLA-G isoform expression, it will be important to analyze, whether HLA-G isoforms are present as monomers, dimers or even as homotrimers (113) associated with or without β_2_-m (20) and with distinct immune suppressive activities. In particular, for the HLA-G dimer an efficient inhibition of CD8^+^ T cells and granzyme B was shown (114). The formation of HLA-G multimers again affects the antibody-based recognition, since some mAbs are detecting the respective HC, but only in complex with the β_2_-m.

In summary, HLA-G has multiple splicing mechanisms leading to different HLA-G isoforms, which are not all detected with commonly used HLA-G antibodies. A recommendation could be to use variable HLA-G antibodies in order to detect most HLA-G isoforms and draw conclusions with clinical parameters. It will also be imperative to understand the function of the different HLA-G domains and subsequently of the HLA-G multimers. These data highlight that the HLA-G protein is an interesting target for therapy based attempts for its downregulation and to target tumor cells in analogy to the anti-PD-L1 mAbs.

## Role of HLA-G for Cancer Immunotherapy

Due to its interactions with numerous immune effector cell populations, the HLA-G neoexpression has rekindled interest as an immune checkpoint inhibitor and suggested as a potential target. In healthy human renal tissues about 47% (+/- 12%) of the immune cells were CD3^+^ T cells, divided in 44% CD4^+^ and 56% CD8^+^ T cells. About 10% of total immune cells were CD14^+^ and CD16^+^ myeloid cells. The frequency of NK and B cells in the kidney epithelium was 18.2% ± 10.5% and 1.4% ± 1.2%, respectively (115).

Furthermore, a large effect of the HLA-G expression significantly influenced on the immune cell infiltration of RCC. When compared to HLA-G-negative RCCs, HLA-G-positive RCCs had a statistically significant higher infiltration of CD3^+^ and CD8^+^ cells and a non-statistically significant higher number of CD4^+^ and CD56^+^ cells. However, the T cell activation markers CD69 and CD25 did not show a statistically significant difference between HLA-G-negative and HLA-G-positive RCC samples (57).

Based on these characteristics, HLA-G is postulated as a novel potential immune checkpoint in different malignancies (29) raising the question how HLA-G expression influences existing cancer immunotherapies including checkpoint inhibitors?

This question is of increasing interest and therefore currently addressed by various research groups independent of urological malignancies (31). This is due to the HLA-G-mediated inhibition of various immune effector cells of the innate and adaptive immune system.

ICPs physiologically protect against an overreaction of the immune system. Tumor cells use ICPs to escape from immune surveillance. ICPs are e.g., the programmed cell death protein 1 (PDCD-1, PD-1) expressed on T cells, its ligand CD274, the programmed cell death protein ligand 1 (PD-L1; B7H1) expressed on tumor cells and immune cells (T cells, B cells, DCs, NKs, macrophages), the cytotoxic T-lymphocyte-associated antigen (CTLA-4) expressed on T cells (T helper cells, cytotoxic T cells and regulatory T cells) and its immunological counterparts B7-1/B7-2 (CD80/CD86) expressed on antigen presenting cells (APCs: DCs, monocytes, macrophages and B lymphocytes). Several ICP inhibitors targeting CTLA4 and the PD1/PD-L1 axes have been recently approved by the EMA and/or the FDA. Their application can result in an activation of different immune cells accompanied by tumor cell elimination, leading to the remarkable success of this therapeutic approach in different cancers including RCC and BC (116–121). Recently, multiple ICPs are investigated as novel targets in experimental tumor models or in clinical trials, like LAG3, TIM-3, TIGIT, BTLA and/or agonists of the co-stimulatory receptors GITR, OX40, 41BB and ICOS (93).

Next to HLA-G and PD-L1 (B7-H1) *in silico* analyses of RCC TCGA data also identified B7-H3, B7-H5, HVEM, CD40, CD70 and ILT2 (on tumor cells) as putative novel ICP axes (122). Indeed, different innovative immunotherapies are in the clinical development for the treatment of patients with RCC. These include inhibitors of ICP, costimulatory agonists, modified cytokines, metabolic modulators, cell therapy and therapeutic vaccines (123).

However, the currently available ICPi are mostly restricted to T cells, since CTLA-4 and PD-1 are expressed on T cells (29). During pregnancy trophoblasts express and secrete members of the B7 family (B7H1/CD274/PD-L1 and B7H3/CD276) as well as HLA-G as immune suppressive proteins (124). Due to the abilities of HLA-G to inhibit various immune effector cell populations including NK cells, CD8^+^ CTL, CD4^+^ T helper cells, B cells and other APC, HLA-G appears to be a potent candidate for further anti-tumor immunotherapy aiming on inhibition of immune tolerance/suppression/evasion exerted by HLA-G expressing tumors.

In numerous *in vitro* studies, the downregulation of HLA-G protein levels, e.g. by overexpression of HLA-G regulatory miRs (63), by HLA-G-specific CRISPR/Cas9 systems (125) or by simple inhibition of HLA-G with antibodies (96), resulted in an increased lysis of tumor cells by immune effector cells. Recently, also chimeric antigen receptors (CARs) directed against HLA-G have been published (126).

Beside the advantages of blocking HLA-G alone or in combination with other immunotherapies including antibodies directed against the checkpoint axes PD-L1/CTLA4 the annual costs per patient receiving ICPi treatment has to be considered. Combination of an anti-HLA-G antibody or antibodies directed against the HLA-G receptors with ICPi might be challenging for financial reasons. The other molecular biological approaches modulating HLA-G expression still requires a long time before successful translation into the clinics, but might offer less expensive alternatives. Another limitation of a possible down-regulation of the HLA-G are putative side effects in regard of the physiological HLA-G expression within the immune privileged tissues. Would a long-term anti-HLA-G therapy increase the risk for irreversible tissue damages by any inflammatory reactions? What are the adverse effects by combining anti-HLA-G therapies with other immunotherapies?

Regarding antibody-based therapies, PD-L1 glycosylation has been shown to lower the binding affinity of respective therapeutic antibodies (avelumab, durvalumab, atezolizumab) (127). It is known that also HLA-G as well as ILT2 can be glycosylated (128, 129), but so far no information exists about their glycosylation pattern in RCCs or BCs, which might be associated with a possible negative effect on the affinity of anti-HLA-G antibodies.

## What are the Functional Consequences of HLA-G Expression for Immune Cells?

Soluble HLA-G released by tumor cells interacts with NK cell receptors and CD8^+^ T cell receptors and even may cause apoptosis of immune effector cells as well as the functional inhibition of immune effector cells. HLA-G suppresses proliferation of CD4^+^ T lymphocytes (130, 131). In addition, sHLA-G alters CD4^+^ and CD8^+^ cells resulting in a loss of their capacity to respond to antigenic stimulation and to their differentiation into immune tolerant Tregs (25). Tregs, DCs and tumor cells can produce and release the anti-inflammatory cytokine interleukin 10 (IL-10), which can promote the expression of HLA-G (29). Membrane-bound HLA-G can affect immune effector cells by trogocytosis, a rapid intercellular transfer of membrane fragments and their associated molecules at intercellular contact (132). In this way, HLA-G can be transferred from tumor cells to activated NK cells or to monocytes. Since transferred HLA-G remains functional, the immune effector cells with acquired HLA-G on their surface do not attack the tumor cells and even gain the capability to inhibit other immune effector cells (29). Beside HLA-G localization on tumor cells, it can also occur in EVs, e.g. in exudates or serum/plasma from cancer patients (133).

## HLA-G and the Immune Cell Infiltration of Tumors

It has been suggested that HLA-G expression can be involved in the immune editing process, which is defined by three distinct stages of immune responses and the interaction between tumor cells with their microenvironment: the elimination, equilibrium and escape (134). During the elimination phase, HLA-G can inhibit T and B cell activation, proliferation, cytotoxic function of T and NK cells as well as DC function. In the equilibrium phase, HLA-G can downregulate MHC class I expression and induce suppressive myeloid cells (MDSC) as well as regulatory T cells (Tregs) (135–137). The escape phase is characterized by an increased cell proliferative rate and a hypoxic environment. HLA-G has been shown to be induced by the hypoxia inducible factor (HIF)-1 and the vascular epidermal growth factor (VEGF) (94). Furthermore, immune suppressive cytokines, such as IL-10 and TGF-β, are often secreted by tumor cells. For example, in non-small cell lung carcinoma (NSCLC), a loss of classical HLA class I antigens was found to be associated with an upregulation of HLA-G as well as IL-10 expression. In ovarian cancer, HLA-G expression correlated with an elevated expression of tumor marker CA-125 and a combination of both serum markers could improve the clinical screening and diagnosis (138). Multiple studies revealed a broad immune regulatory role of HLA-G affecting innate and adaptive immune responses. Overall, the immune inhibitory mechanisms mediated by HLA-G can be summarized in three main categories: (i) direct inhibition of effector cells and antigen presenting cells, (ii) indirect immune inhibition through induction of regulatory cells and (iii) other mechanisms.

Interestingly, CD8^+^ ILT2^+^ T cells in the TME (tumor infiltrating lymphocytes, TIL) show a more mature and aggressive CTL phenotype with a higher cytolytic capacity compared to ILT2-negative peripheral blood precursors or CD8^+^ PD-1^+^ TIL. Since HLA-G is able to nearly completely block their activity, this cell pool is an interesting target to release their cytolytic capacity by therapeutic HLA-G inhibition in HLA-G expressing tumors (139). This observation might be particularly interesting for cancer entities, where HLA-G neoexpression has been associated with concomitant high immune infiltration levels, such as Ewing sarcoma and RCC (140).

Indirect immunosuppressive mechanisms are usually based on the induction of durable immune suppressive effects through the generation or induction of Tregs and/or accumulation of myeloid suppressor cells (135). HLA-G is able to impair T helper cell alloproliferation, the regular function of CD4^+^ T cells and to induce their differentiation into Tregs to indirectly increase immunosuppressive effects in the TME (141). Interestingly, HLA-G-induced Tregs are dependent on HLA-G during the differentiation, but not for their immunosuppressive function (142). Transient immunosuppressive effects *via* HLA-G can also be mediated by NK cells, CTLs and monocytes/macrophages, which acquired HLA-G expressing membrane components from other cells *via* trogocytosis (132). Furthermore, HLA-G mediates immune suppression *via* so called “DC-10” dendritic cells (dendritic cells characterized by IL-10 production), which are characterized by high expression of HLA-G and other tolerogenic molecules, such as ILT2 and ILT4 (143). DC-10 cells are known as potent stimulators of allospecific type 1 Tregs, which play a crucial role in promoting and maintaining durable immune tolerance and immune suppression (58, 143). Other immune suppressive mechanisms of HLA-G include the induction of apoptosis of effector cell populations *via* sHLA-G, upregulation of immune inhibitory receptors (including KIR2DL4, ILT2 and ILT4), the suppression of IFN-ƴ release by NK cells (131, 144–147) and inhibition of NK cells and CTL *via* indirect induction of HLA-E expression, which can activate the inhibitory immune checkpoint NKG2A expressed on T and NK cells (148, 149). Furthermore, the HLA-G expression may be induced after infection with human papilloma viruses (150), which are known to have moderate effects on urological malignancies including RCCs and BCs (151).

## Conclusions

**Figure 2:**
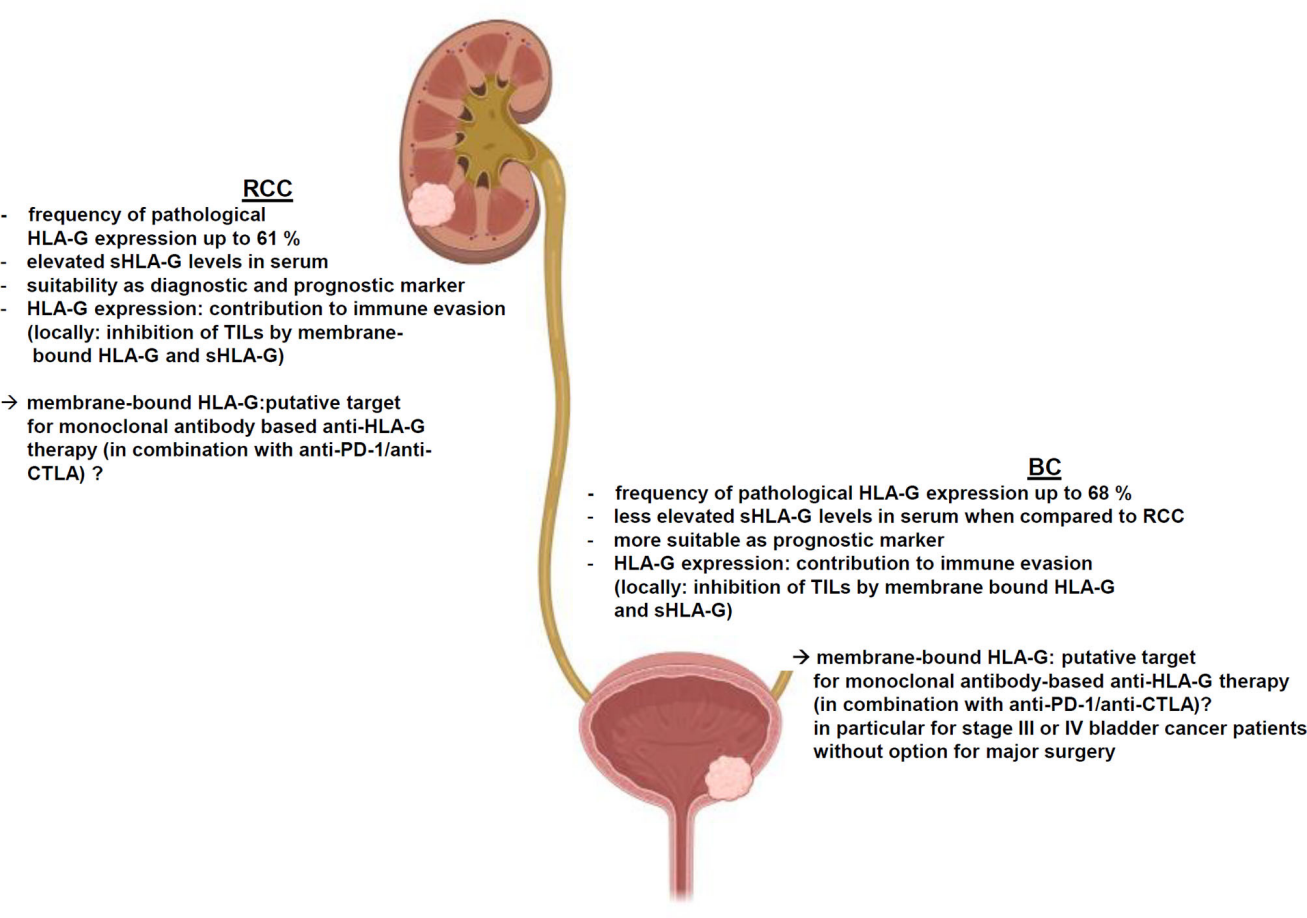
HLA-G-mediated immune escape of RCC and bladder cancer and its clinical relevance.

HLA-G is frequently, but heterogeneously expressed in both RCC and BC, which is dependent on the tumor subtype, tumor grading or staging as well as the composition of the immune cell infiltration. Unlike other regulatory ICPs, HLA-G exhibits its immune regulatory and immune suppressive functions at multiple levels of the immune response and it is able to either inhibit or stimulate key immune cell populations involved in immune responses to induce potent long-term immune suppression. Despite little information exists regarding the functional link between HLA-G expression and immune responses, an impaired NK cell- and CTL-mediated recognition of HLA-G-expressing RCC cell lines has been shown, but deserves further investigations in RCC and BC. As summarized in **Figure 2**, HLA-G contributes to the immune escape of both RCC and BC by inhibiting TIL activity due to its high frequency of expression and clinical relevance in both diseases. However, the immune regulatory and immune suppressive functions of HLA-G are considered to be much more profound and complex than those of individual co-inhibitory ICPs, such as PD-L1, CTLA-4 or PD-1, which currently serve as common targets for clinically approved immunotherapies. Recent knowledge offers insights into the underlying molecular mechanisms of HLA-G neoexpression demonstrating a role of HLA-G regulatory miRNAs in RCC. Furthermore, the TME consisting of immune suppressive cytokines secreted by either RCC and BC cells or by different immune cells might impair immune effector responses. In this context, HLA-G might serve as diagnostic and/or prognostic marker or as novel therapeutic target for both malignancies.

Therefore, investigations are urgently required to monitor membranous and sHLA-G in both malignancies in general, but also during immunotherapies of RCC and BC. This will give insights into the potential of HLA-G to serve as target for diverse immunotherapies of HLA-G-expressing tumors than singular inhibition of a less significant ICP – especially in the view of combination therapies.

## Author Contributions

BS and SJ-B designed the study. BS, SJ-B, ME, HT, SW, CF, RS, and AH wrote the manuscript. All authors contributed to the article and approved the submitted version.

## Funding

This work was funded by the German-Israeli Foundation (GIF; I-37-414.11-2016) and Dr. Werner Jackstädt Foundation.

## Conflict of Interest

The authors declare that the research was conducted in the absence of any commercial or financial relationships that could be construed as a potential conflict of interest.

## Publisher’s Note

All claims expressed in this article are solely those of the authors and do not necessarily represent those of their affiliated organizations, or those of the publisher, the editors and the reviewers. Any product that may be evaluated in this article, or claim that may be made by its manufacturer, is not guaranteed or endorsed by the publisher.

## Abbreviations

AIRE, autoimmune regulator; APC, antigen presenting cell; APM, antigen processing machinery; β2-m, β2-microglobulin; BC, bladder carcinoma; BCG, Bacillus Calmette-Guerin; bp, base pair; CAR, chimeric antigen receptor; ccRCC, clear cell renal cell carcinoma; CRC, colorectal carcinoma; CREP1, cAMP-responsive element binding protein 1; CTL, cytotoxic T lymphocyte; CTLA4, cytotoxic T lymphocyte-associated protein-4; DC, dendritic cell; EV, extracellular vesicle; HC, heavy chain; HIF, hypoxia inducible factor; HLA, human leukocyte antigen; ICP, immune checkpoint; ICP-L, immune checkpoint ligand; ICP-R, immune checkpoint receptor; IFN, interferon; IHC, immunohistochemistry; ILT, immunoglobulin-like transcript; IL-10; LAG3, lymphocyte activation gene 3; mAb, monoclonal antibody; MHC, major histocompatibility complex; miRNA, microRNA; MMP2, matrix metalloproteinase 2; NK, natural killer; OS, overall survival; PD1, programmed cell death-1; PD-L1, programmed death ligand 1; PFS, progression-free survival; RBP, RNA-binding protein; RCC, renal cell carcinoma; sHLA-G, soluble HLA-G; TAP, transporter associated with antigen processing; TGF-β, transforming growth factor β; TIGIT, T cell immune receptor with immunoglobulin and ITIM domain; TIL, tumor infiltrating lymphocyte; TIM-3, T cell immunoglobulin and mucin domain 3; TM, transmembrane; TME, tumor microenvironment; Treg, regulatory T cell; UTR, untranslated region; VEGF, vascular endothelial growth factor; VHL, Von Hippel-Lindau; VISTA, V-domain Ig suppressor of T cell activation; WB, Western blot.

## References

[1] Galon J Bruni D. Tumor Immunology and Tumor Evolution: Intertwined Histories. Immunity (2020) 52(1):55–81. doi: 10.1016/j.immuni.2019.12.018 31940273

[2] Saxena S Singh RK. Chemokines Orchestrate Tumor Cells and the Microenvironment to Achieve Metastatic Heterogeneity. Cancer Metastasis Rev (2021) 40(2):447–76. doi: 10.1007/s10555-021-09970-6 PMC9863248 33959849

[3] Kaymak I Williams KS Cantor JR Jones RG. Immunometabolic Interplay in the Tumor Microenvironment. Cancer Cell (2021) 39(1):28–37. doi: 10.1016/j.ccell.2020.09.004 33125860 PMC7837268

[4] Elia I Haigis MC. Metabolites and the Tumour Microenvironment: From Cellular Mechanisms to Systemic Metabolism. Nat Metab (2021) 3(1):21–32. doi: 10.1038/s42255-020-00317-z 33398194 PMC8097259

[5] Joller N Kuchroo VK. Tim-3, Lag-3, and TIGIT. Curr Top Microbiol Immunol (2017) 410:127–56. doi: 10.1007/82_2017_62 PMC5902028 28900677

[6] Lee JB Ha SJ Kim HR. Clinical Insights Into Novel Immune Checkpoint Inhibitors. Front Pharmacol (2021) 12:681320. doi: 10.3389/fphar.2021.681320 34025438 PMC8139127

[7] Dhatchinamoorthy K Colbert JD Rock KL. Cancer Immune Evasion Through Loss of MHC Class I Antigen Presentation. Front Immunol (2021) 12:636568. doi: 10.3389/fimmu.2021.636568 33767702 PMC7986854

[8] Chen DS Mellman I. Elements of Cancer Immunity and the Cancer-Immune Set Point. Nature (2017) 541(7637):321–30. doi: 10.1038/nature21349 28102259

[9] Vinay DS Ryan EP Pawelec G Talib WH Stagg J Elkord E. Immune Evasion in Cancer: Mechanistic Basis and Therapeutic Strategies. Semin Cancer Biol (2015) 35(Suppl):S185–98. doi: 10.1016/j.semcancer.2015.03.004 25818339

[10] Duan Q Zhang H Zheng J Zhang L. Turning Cold Into Hot: Firing Up the Tumor Microenvironment. Trends Cancer (2020) 6(7):605–18. doi: 10.1016/j.trecan.2020.02.022 32610070

[11] Zhou R Caspi RR. Ocular Immune Privilege. F1000 Biol Rep (2010) 2. doi: 10.3410/B2-3 PMC2948372 20948803

[12] Le Discorde M Moreau P Sabatier P Legeais J Carosella ED. Expression of HLA-G in Human Cornea, an Immune-Privileged Tissue. Hum Immunol (2003) 64(11):1039–44. doi: 10.1016/j.humimm.2003.08.346 14602233

[13] Higa K Shimmura S Shimazaki J Tsubota K. Ocular Surface Epithelial Cells Up-Regulate HLA-G When Expanded In Vitro on Amniotic Membrane Substrates. Cornea (2006) 25(6):715–21. doi: 10.1097/01.ico.0000214227.36485.9b 17077667

[14] Svendsen SG Søberg Udsen M Daouya M Funck T Wu C Carosella ED. Expression and Differential Regulation of HLA-G Isoforms in the Retinal Pigment Epithelial Cell Line, ARPE-19. Hum Immunol (2017) 78(5-6):414–20. doi: 10.1016/j.humimm.2017.04.007 28442288

[15] Cirulli V Zalatan J McMaster M Prinsen R Salomon DR Ricordi C. The Class I HLA Repertoire of Pancreatic Islets Comprises the Nonclassical Class Ib Antigen HLA-G. Diabetes (2006) 55(5):1214–22. doi: 10.2337/db05-0731 16644675

[16] Mallet V Blaschitz A Crisa L Schmitt C Fournel S King A. HLA-G in the Human Thymus: A Subpopulation of Medullary Epithelial But Not CD83(+) Dendritic Cells Expresses HLA-G as a Membrane-Bound and Soluble Protein. Int Immunol (1999) 11(6):889–98. doi: 10.1093/intimm/11.6.889 10360962

[17] McMaster MT Librach CL Zhou Y Lim KH Janatpour MJ DeMars R. Human Placental HLA-G Expression Is Restricted to Differentiated Cytotrophoblasts. J Immunol (1995) 154(8):3771–8. 7706718

[18] Kovats S Main EK Librach C Stubblebine M Fisher SJ DeMars R. A Class I Antigen, HLA-G, Expressed in Human Trophoblasts. Science (1990) 248(4952):220–3. doi: 10.1126/science.2326636 2326636

[19] Ellis SA Palmer MS McMichael AJ. Human Trophoblast and the Choriocarcinoma Cell Line BeWo Express a Truncated HLA Class I Molecule. J Immunol (1990) 144(2):731–5. 2295808

[20] Carosella ED Favier B Rouas-Freiss N Moreau P Lemaoult J. Beyond the Increasing Complexity of the Immunomodulatory HLA-G Molecule. Blood (2008) 111(10):4862–70. doi: 10.1182/blood-2007-12-127662 18334671

[21] Carosella ED Gregori S LeMaoult J. The Tolerogenic Interplay(s) Among HLA-G, Myeloid APCs, and Regulatory Cells. Blood (2011) 118(25):6499–505. doi: 10.1182/blood-2011-07-370742 21960588

[22] Rouas-Freiss N Moreau P LeMaoult J Papp B Tronik-Le Roux D Carosella ED. Role of the HLA-G Immune Checkpoint Molecule in Pregnancy. Hum Immunol (2021) 82(5):353–61. doi: 10.1016/j.humimm.2021.01.003 33745758

[23] Friedrich M Jasinski-Bergner S Lazaridou M Subbarayan K Massa C Tretbar S. Tumor-Induced Escape Mechanisms and Their Association With Resistance to Checkpoint Inhibitor Therapy. Cancer Immunol Immunother (2019) 68(10):1689–700. doi: 10.1007/s00262-019-02373-1 PMC11028168 31375885

[24] Rouas-Freiss N Moreau P Ferrone S Carosella ED. HLA-G Proteins in Cancer: Do They Provide Tumor Cells With an Escape Mechanism? Cancer Res (2005) 65(22):10139–44. doi: 10.1158/0008-5472.CAN-05-0097 16287995

[25] Curigliano G Criscitiello C Gelao L Goldhirsch A. Molecular Pathways: Human Leukocyte Antigen G (HLA-G). Clin Cancer Res (2013) 19(20):5564–71. doi: 10.1158/1078-0432.CCR-12-3697 23897901

[26] Attia JVD Dessens CE van de Water R Houvast RD Kuppen PJK Krijgsman D. The Molecular and Functional Characteristics of HLA-G and the Interaction With Its Receptors: Where to Intervene for Cancer Immunotherapy? Int J Mol Sci (2020) 21(22):8678. doi: 10.3390/ijms21228678 PMC7698525 33213057

[27] Lin A Yan WH. HLA-G/ILTs Targeted Solid Cancer Immunotherapy: Opportunities and Challenges. Front Immunol (2021) 12:698677. doi: 10.3389/fimmu.2021.698677 34276691 PMC8278316

[28] Le Friec G Gros F Sebti Y Guilloux V Pangault C Fauchet R. Capacity of Myeloid and Plasmacytoid Dendritic Cells Especially at Mature Stage to Express and Secrete HLA-G Molecules. J Leukoc Biol (2004) 76(6):1125–33. doi: 10.1189/jlb.0104015 15331623

[29] Carosella ED Rouas-Freiss N Tronik-Le Roux D Moreau P LeMaoult J. HLA-G: An Immune Checkpoint Molecule. Adv Immunol (2015) 127:33–144. doi: 10.1016/bs.ai.2015.04.001 26073983

[30] Selmani Z Naji A Gaiffe E Obert L Tiberghien P Rouas-Freiss N. HLA-G Is a Crucial Immunosuppressive Molecule Secreted by Adult Human Mesenchymal Stem Cells. Transplantation (2009) 87(9 Suppl):S62–6. doi: 10.1097/TP.0b013e3181a2a4b3 19424010

[31] Carosella ED Gregori S Tronik-Le Roux D. HLA-G/LILRBs: A Cancer Immunotherapy Challenge. Trends Cancer (2021) 7(5):389–92. doi: 10.1016/j.trecan.2021.01.004 33563576

[32] Robinson J Halliwell JA Hayhurst JD Flicek P Parham P Marsh SGE. The IPD and IMGT/HLA Database: Allele Variant Databases. Nucleic Acids Res (2015) 43(Database issue):D423–31. doi: 10.1093/nar/gku1161 PMC4383959 25414341

[33] Hviid TV. HLA-G in Human Reproduction: Aspects of Genetics, Function and Pregnancy Complications. Hum Reprod Update (2006) 12(3):209–32. doi: 10.1093/humupd/dmi048 16280356

[34] Ishitani A Geraghty DE. Alternative Splicing of HLA-G Transcripts Yields Proteins With Primary Structures Resembling Both Class I and Class II Antigens. Proc Natl Acad Sci USA (1992) 89(9):3947–51. doi: 10.1073/pnas.89.9.3947 PMC525608 1570318

[35] Fujii T Ishitani A Geraghty DE. A Soluble Form of the HLA-G Antigen Is Encoded by a Messenger Ribonucleic Acid Containing Intron 4. J Immunol (1994) 153(12):5516–24. 7989753

[36] Tronik-Le Roux D Renard J Vérine J Renault V Tubacher E LeMaoult J. Novel Landscape of HLA-G Isoforms Expressed in Clear Cell Renal Cell Carcinoma Patients. Mol Oncol (2017) 11(11):1561–78. doi: 10.1002/1878-0261.12119 PMC5664004 28815885

[37] Lin A Yan WH. Heterogeneity of HLA-G Expression in Cancers: Facing the Challenges. Front Immunol (2018) 9:2164. doi: 10.3389/fimmu.2018.02164 30319626 PMC6170620

[38] d'Almeida TC Sadissou I Milet J Cottrell G Mondière A Avokpaho E. Soluble Human Leukocyte Antigen -G During Pregnancy and Infancy in Benin: Mother/child Resemblance and Association With the Risk of Malaria Infection and Low Birth Weight. PloS One (2017) 12(2):e0171117. doi: 10.1371/journal.pone.0171117 28166246 PMC5293225

[39] Munz C Stevanovic S Rammensee HG. Peptide Presentation and NK Inhibition by HLA-G. J Reprod Immunol (1999) 43(2):139–55. doi: 10.1016/S0165-0378(99)00029-7 10479050

[40] Park GM Lee S Park B Kim E Shin J Cho K. Soluble HLA-G Generated by Proteolytic Shedding Inhibits NK-Mediated Cell Lysis. Biochem Biophys Res Commun (2004) 313(3):606–11. doi: 10.1016/j.bbrc.2003.11.153 14697234

[41] Rizzo R Trentini A Bortolotti D Manfrinato MC Rotola A Castellazzi M. Matrix Metalloproteinase-2 (MMP-2) Generates Soluble HLA-G1 by Cell Surface Proteolytic Shedding. Mol Cell Biochem (2013) 381(1-2):243–55. doi: 10.1007/s11010-013-1708-5 23737137

[42] Krijgsman D Roelands J Hendrickx W Bedognetti D Kuppen PJK. HLA-G: A New Immune Checkpoint in Cancer? Int J Mol Sci (2020) 21(12):4528. doi: 10.3390/ijms21124528 PMC7350262 32630545

[43] Dunker K Schlaf G Bukur J Altermann WW Handke D Seliger B. Expression and Regulation of Non-Classical HLA-G in Renal Cell Carcinoma. Tissue Antigens (2008) 72(2):137–48. doi: 10.1111/j.1399-0039.2008.01090.x 18721274

[44] Moreau P Mouillot G Rousseau P Marcou C Dausset J Carosella ED. HLA-G Gene Repression Is Reversed by Demethylation. Proc Natl Acad Sci USA (2003) 100(3):1191–6. doi: 10.1073/pnas.0337539100 PMC298749 12552087

[45] Schiano C Benincasa G Infante T Franzese M Castaldo R Fiorito C. Integrated Analysis of DNA Methylation Profile of HLA-G Gene and Imaging in Coronary Heart Disease: Pilot Study. PloS One (2020) 15(8):e0236951. doi: 10.1371/journal.pone.0236951 32790754 PMC7425923

[46] Tang Y Liu H Li H Peng T Gu W Li X. Hypermethylation of the HLA-G Promoter Is Associated With Preeclampsia. Mol Hum Reprod (2015) 21(9):736–44. doi: 10.1093/molehr/gav037 26116450

[47] Mouillot G Marcou C Rousseau P Rouas-Freiss N Carosella ED Moreau P. HLA-G Gene Activation in Tumor Cells Involves Cis-Acting Epigenetic Changes. Int J Cancer (2005) 113(6):928–36. doi: 10.1002/ijc.20682 15514928

[48] Melo-Lima BL Poras I Passos GA Carosella ED Donadi EA Moreau P. The Autoimmune Regulator (Aire) Transactivates HLA-G Gene Expression in Thymic Epithelial Cells. Immunology (2019) 158(2):121–35. doi: 10.1111/imm.13099 PMC6742766 31322727

[49] Perniola R. Twenty Years of AIRE. Front Immunol (2018) 9:98. doi: 10.3389/fimmu.2018.00098 29483906 PMC5816566

[50] Cepeda S Cantu C Orozco S Xiao Y Brown Z Semwal MK. Age-Associated Decline in Thymic B Cell Expression of Aire and Aire-Dependent Self-Antigens. Cell Rep (2018) 22(5):1276–87. doi: 10.1016/j.celrep.2018.01.015 PMC5813500 29386114

[51] Gobin SJ Biesta P de Steenwinkel JEM Datema G van den Elsen PJ. HLA-G Transactivation by cAMP-Response Element-Binding Protein (CREB). An Alternative Transactivation Pathway to the Conserved Major Histocompatibility Complex (MHC) Class I Regulatory Routes. J Biol Chem (2002) 277(42):39525–31. doi: 10.1074/jbc.M112273200 12183445

[52] Friedrich M Stoehr C Jasinski-Bergner S Hartmann A Wach S Wullich B. Characterization of the Expression and Immunological Impact of the Transcriptional Activator CREB in Renal Cell Carcinoma. J Transl Med (2020) 18(1):371. doi: 10.1186/s12967-020-02544-0 32993793 PMC7526213

[53] Lefebvre S Berrih-Aknin S Adrian F Moreau P Poea S Gourand L. A Specific Interferon (IFN)-Stimulated Response Element of the Distal HLA-G Promoter Binds IFN-Regulatory Factor 1 and Mediates Enhancement of This Nonclassical Class I Gene by IFN-Beta. J Biol Chem (2001) 276(9):6133–9. doi: 10.1074/jbc.M008496200 11087747

[54] Flajollet S Poras I Carosella ED Moreau P. RREB-1 Is a Transcriptional Repressor of HLA-G. J Immunol (2009) 183(11):6948–59. doi: 10.4049/jimmunol.0902053 19890057

[55] Ikeno M Suzuki N Kamiya M Takahashi Y Kudoh J Okazaki T. LINE1 Family Member Is Negative Regulator of HLA-G Expression. Nucleic Acids Res (2012) 40(21):10742–52. doi: 10.1093/nar/gks874 PMC3510505 23002136

[56] Amiot L Vu N Samson M. Immunomodulatory Properties of HLA-G in Infectious Diseases. J Immunol Res (2014) 2014:298569. doi: 10.1155/2014/298569 24839609 PMC4009271

[57] Jasinski-Bergner S Stoehr C Bukur J Massa C Braun J Hüttelmaier S. Clinical Relevance of miR-Mediated HLA-G Regulation and the Associated Immune Cell Infiltration in Renal Cell Carcinoma. Oncoimmunology (2015) 4(6):e1008805. doi: 10.1080/2162402X.2015.1008805 26155421 PMC4485830

[58] Roncarolo MG Gregori S Bacchetta R Battaglia M Gagliani N. The Biology of T Regulatory Type 1 Cells and Their Therapeutic Application in Immune-Mediated Diseases. Immunity (2018) 49(6):1004–19. doi: 10.1016/j.immuni.2018.12.001 30566879

[59] Seliger B. Role of microRNAs on HLA-G Expression in Human Tumors. Hum Immunol (2016) 77(9):760–3. doi: 10.1016/j.humimm.2016.04.006 27142884

[60] Reches A Nachmani D Berhani O Duev-Cohen A Shreibman D Ophir Y. HNRNPR Regulates the Expression of Classical and Nonclassical MHC Class I Proteins. J Immunol (2016) 196(12):4967–76. doi: 10.4049/jimmunol.1501550 27194785

[61] Sun J Chu H Ji J Huo G Song Q Zhang X. Long Non-Coding RNA HOTAIR Modulates HLA-G Expression by Absorbing miR-148a in Human Cervical Cancer. Int J Oncol (2016) 49(3):943–52. doi: 10.3892/ijo.2016.3589 27574106

[62] Manaster I Goldman-Wohl D Greenfield C Nachmani D Tsukerman P Hamani Y. MiRNA-Mediated Control of HLA-G Expression and Function. PloS One (2012) 7(3):e33395. doi: 10.1371/journal.pone.0033395 22438923 PMC3306401

[63] Jasinski-Bergner S Reches A Stoehr C Massa C Gonschorek E Huettelmaier S. Identification of Novel microRNAs Regulating HLA-G Expression and Investigating Their Clinical Relevance in Renal Cell Carcinoma. Oncotarget (2016) 7(18):26866–78. doi: 10.18632/oncotarget.8567 PMC5042021 27057628

[64] Friedrich M Vaxevanis CK Biehl K Mueller A Seliger B. Targeting the Coding Sequence: Opposing Roles in Regulating Classical and Non-Classical MHC Class I Molecules by miR-16 and miR-744. J Immunother Cancer (2020) 8(1):e000396. doi: 10.1136/jitc-2019-000396 32571994 PMC7307530

[65] Mori A Nishi H Sasaki T Nagamitsu Y Kawaguchi R Okamoto A. HLA-G Expression Is Regulated by miR-365 in Trophoblasts Under Hypoxic Conditions. Placenta (2016) 45:37–41. doi: 10.1016/j.placenta.2016.07.004 27577708

[66] Wang X Li B Wang J Lei J Liu C Ma Y. Evidence That miR-133a Causes Recurrent Spontaneous Abortion by Reducing HLA-G Expression. Reprod BioMed Online (2012) 25(4):415–24. doi: 10.1016/j.rbmo.2012.06.022 22877943

[67] Castelli EC Mendes-Junior CT Deghaide NHS de Albuquerque RS Muniz YCN Simões RT. The Genetic Structure of 3'untranslated Region of the HLA-G Gene: Polymorphisms and Haplotypes. Genes Immun (2010) 11(2):134–41. doi: 10.1038/gene.2009.74 19798077

[68] Reches A Berhani O Mandelboim O. A Unique Regulation Region in the 3' UTR of HLA-G With a Promising Potential. Int J Mol Sci (2020) 21(3):900. doi: 10.3390/ijms21030900 PMC7037441 32019184

[69] Song B Guan Z Liu F Sun D Wang K Qu H. Long Non-Coding RNA HOTAIR Promotes HLA-G Expression via Inhibiting miR-152 in Gastric Cancer Cells. Biochem Biophys Res Commun (2015) 464(3):807–13. doi: 10.1016/j.bbrc.2015.07.040 26187665

[70] Bukur J Jasinski S Seliger B. The Role of Classical and Non-Classical HLA Class I Antigens in Human Tumors. Semin Cancer Biol (2012) 22(4):350–8. doi: 10.1016/j.semcancer.2012.03.003 22465194

[71] He X Dong D Yie S Yang H Cao M Ye S. HLA-G Expression in Human Breast Cancer: Implications for Diagnosis and Prognosis, and Effect on Allocytotoxic Lymphocyte Response After Hormone Treatment In Vitro. Ann Surg Oncol (2010) 17(5):1459–69. doi: 10.1245/s10434-009-0891-9 20052552

[72] de Kruijf EM Sajet A van Nes JGH Natanov R Putter H Smit VTHBM. HLA-E and HLA-G Expression in Classical HLA Class I-Negative Tumors Is of Prognostic Value for Clinical Outcome of Early Breast Cancer Patients. J Immunol (2010) 185(12):7452–9. doi: 10.4049/jimmunol.1002629 21057081

[73] Li XJ Zhang X Lin A Ruan Y Yan W. Human Leukocyte Antigen-G (HLA-G) Expression in Cervical Cancer Lesions Is Associated With Disease Progression. Hum Immunol (2012) 73(9):946–9. doi: 10.1016/j.humimm.2012.07.041 22820627

[74] Zheng N Wang C Zhang X Du L Zhang J Kan S. Up-Regulation of HLA-G Expression in Cervical Premalignant and Malignant Lesions. Tissue Antigens (2011) 77(3):218–24. doi: 10.1111/j.1399-0039.2010.01607.x 21299526

[75] Zhang RL Zhang X Dong S Hu B Han Q Zhang J. Predictive Value of Different Proportion of Lesion HLA-G Expression in Colorectal Cancer. Oncotarget (2017) 8(64):107441–51. doi: 10.18632/oncotarget.22487 PMC5746078 29296176

[76] Zhu CB Wang C Zhang X Zhang J Li W. Serum sHLA-G Levels: A Useful Indicator in Distinguishing Colorectal Cancer From Benign Colorectal Diseases. Int J Cancer (2011) 128(3):617–22. doi: 10.1002/ijc.25372 20473865

[77] Murdaca G Calamaro P Lantieri F Pigozzi S Mastracci L Grillo F. HLA-G Expression in Gastric Carcinoma: Clinicopathological Correlations and Prognostic Impact. Virchows Arch (2018) 473(4):425–33. doi: 10.1007/s00428-018-2379-0 29845360

[78] Wastowski IJ Simões RT Yaghi L Donadi EA Pancoto JT Poras I. Human Leukocyte Antigen-G Is Frequently Expressed in Glioblastoma and may be Induced In Vitro by Combined 5-Aza-2'-Deoxycytidine and Interferon-Gamma Treatments: Results From a Multicentric Study. Am J Pathol (2013) 182(2):540–52. doi: 10.1016/j.ajpath.2012.10.021 PMC3562737 23219427

[79] Khodabandeh Shahraki P Zare Y Azarpira N Hosseinzadeh M Farjadian S. Prognostic Value of HLA-G in Malignant Liver and Pancreas Lesions. Iran J Immunol (2018) 15(1):28–37. 29549230 10.22034/iji.2018.39338

[80] Rutten MJ Dijk F Savci-Heijink CD Buist MR Kenter GG van de Vijver MJ. HLA-G Expression Is an Independent Predictor for Improved Survival in High Grade Ovarian Carcinomas. J Immunol Res (2014) 2014:274584. doi: 10.1155/2014/274584 24987709 PMC4058481

[81] Lin A Yan W Xu H Gan M Cai J Zhu M. HLA-G Expression in Human Ovarian Carcinoma Counteracts NK Cell Function. Ann Oncol (2007) 18(11):1804–9. doi: 10.1093/annonc/mdm356 17846022

[82] Karagoz B Haholu A Ozgün A Bilgi O Tunçel T Emirzeoğlu L. HLA-G in Testicular Germ Cell Tumors. Oncol Res Treat (2014) 37(5):245–8. doi: 10.1159/000362377 24853783

[83] Caocci G Greco M Arras M Cusano R Orrù S Martino B. HLA-G Molecules and Clinical Outcome in Chronic Myeloid Leukemia. Leuk Res (2017) 61:1–5. doi: 10.1016/j.leukres.2017.08.005 28841441

[84] Ozet G Falay M Dagdas S Ceran F. Determination of HLA-G Expression and Evaluation of Its Role as a Prognostic Factor in Chronic Lymphocytic Leukemia. J Clin Lab Anal (2016) 30(5):399–403. doi: 10.1002/jcla.21868 26303056 PMC6807200

[85] Schwich E Hò GT LeMaoult J Bade-Döding C Carosella ED Horn PA. Soluble HLA-G and HLA-G Bearing Extracellular Vesicles Affect ILT-2 Positive and ILT-2 Negative CD8 T Cells Complementary. Front Immunol (2020) 11:2046. doi: 10.3389/fimmu.2020.02046 32973812 PMC7472666

[86] Rebmann V König L da Silva Nardi F Wagner B Santos Manvailer LF Horn PA. The Potential of HLA-G-Bearing Extracellular Vesicles as a Future Element in HLA-G Immune Biology. Front Immunol (2016) 7:173. doi: 10.3389/fimmu.2016.00173 27199995 PMC4854879

[87] Amodio G Gregori S. The Discovery of HLA-G-Bearing Extracellular Vesicles: New Perspectives in HLA-G Biology. Ann Transl Med (2017) 5(6):148. doi: 10.21037/atm.2017.01.46 28462228 PMC5395469

[88] Hsieh JJ Purdue MP Signoretti S Swanton C Albiges L Schmidinger M. Renal Cell Carcinoma. Nat Rev Dis Primers (2017) 3:17009. doi: 10.1038/nrdp.2017.9 28276433 PMC5936048

[89] Muglia VF Prando A. Renal Cell Carcinoma: Histological Classification and Correlation With Imaging Findings. Radiol Bras (2015) 48(3):166–74. doi: 10.1590/0100-3984.2013.1927 PMC4492569 26185343

[90] Znaor A Lortet-Tieulent J Laversanne M Jemal A Bray F. International Variations and Trends in Renal Cell Carcinoma Incidence and Mortality. Eur Urol (2015) 67(3):519–30. doi: 10.1016/j.eururo.2014.10.002 25449206

[91] Fan C Zhao C Wang F Li S Wang J. Significance of PTEN Mutation in Cellular Process, Prognosis, and Drug Selection in Clear Cell Renal Cell Carcinoma. Front Oncol (2019) 9:357. doi: 10.3389/fonc.2019.00357 31139560 PMC6518664

[92] Law TM Motzer RJ Mazumdar M Sell KW Walther PJ O'Connell M. Phase III Randomized Trial of Interleukin-2 With or Without Lymphokine-Activated Killer Cells in the Treatment of Patients With Advanced Renal Cell Carcinoma. Cancer (1995) 76(5):824–32. doi: 10.1002/1097-0142(19950901)76:5<824::AID-CNCR2820760517>3.0.CO;2-N 8625186

[93] Kraehenbuehl L Weng C Eghbali S Wolchok JD Merghoub T. Enhancing Immunotherapy in Cancer by Targeting Emerging Immunomodulatory Pathways. Nat Rev Clin Oncol (2021) 19(1):37–50. doi: 10.1038/s41571-021-00552-7 34580473

[94] Garcia M Belen Palma M Verine J Miriuka S Inda AM Errecalde AL. The Immune-Checkpoint HLA-G/ILT4 Is Involved in the Regulation of VEGF Expression in Clear Cell Renal Cell Carcinoma. BMC Cancer (2020) 20(1):624. doi: 10.1186/s12885-020-07113-8 32620162 PMC7333411

[95] Ibrahim EC Guerra N Lacombe MJ Angevin E Chouaib S Carosella ED. Tumor-Specific Up-Regulation of the Nonclassical Class I HLA-G Antigen Expression in Renal Carcinoma. Cancer Res (2001) 61(18):6838–45. 11559559

[96] Bukur J Rebmann V Grosse-Wilde H Luboldt H Ruebben H Drexler I. Functional Role of Human Leukocyte Antigen-G Up-Regulation in Renal Cell Carcinoma. Cancer Res (2003) 63(14):4107–11. 12874014

[97] Li BL Lin A Zhang X Zhang X Zhang J Wang Q. Characterization of HLA-G Expression in Renal Cell Carcinoma. Tissue Antigens (2009) 74(3):213–21. doi: 10.1111/j.1399-0039.2009.01302.x 19531101

[98] Castelli EC Veiga-Castelli LC Yaghi L Moreau P Donadi EA. Transcriptional and Posttranscriptional Regulations of the HLA-G Gene. J Immunol Res (2014) 2014:734068. doi: 10.1155/2014/734068 24741620 PMC3987962

[99] Burger M Catto JWF Dalbagni G Barton Grossman H Herr H Karakiewicz P. Epidemiology and Risk Factors of Urothelial Bladder Cancer. Eur Urol (2013) 63(2):234–41. doi: 10.1016/j.eururo.2012.07.033 22877502

[100] Yang Z Xu Y Bi Y Zhang N Wang H Xing T. Immune Escape Mechanisms and Immunotherapy of Urothelial Bladder Cancer. J Clin Transl Res (2021) 7(4):485–500. 34541363 PMC8445627

[101] Morsch R Rose M Maurer A Cassataro MA Braunschweig T Knüchel R. Therapeutic Implications of PD-L1 Expression in Bladder Cancer With Squamous Differentiation. BMC Cancer (2020) 20(1):230. doi: 10.1186/s12885-020-06727-2 32188412 PMC7079494

[102] Pfannstiel C Strissel PL Chiappinelli KB Sikic D Wach S Wirtz RM. The Tumor Immune Microenvironment Drives a Prognostic Relevance That Correlates With Bladder Cancer Subtypes. Cancer Immunol Res (2019) 7(6):923–38. doi: 10.1158/2326-6066.CIR-18-0758 30988029

[103] Gan LH Huang L Zhang X Lin A Xu D Wang Q. Tumor-Specific Upregulation of Human Leukocyte Antigen-G Expression in Bladder Transitional Cell Carcinoma. Hum Immunol (2010) 71(9):899–904. doi: 10.1016/j.humimm.2010.06.012 20600448

[104] El-Chennawi FA Auf FA El-Diasty AM Abd El-Daim M El-Sherbiny SM Ali A. Expression of HLA-G in Cancer Bladder. Egypt J Immunol (2005) 12(1):57–64. 16734140

[105] Saban MR Hellmich HL Simpson C Davis CA Lang ML Ihnat MA. Repeated BCG Treatment of Mouse Bladder Selectively Stimulates Small GTPases and HLA Antigens and Inhibits Single-Spanning Uroplakins. BMC Cancer (2007) 7:204. doi: 10.1186/1471-2407-7-204 17980030 PMC2212656

[106] Wu CL Svendsen SG Riviere A Desgrandchamps F Carosella ED LeMaoult J. Multiplex Bead-Based Immunoassay for the Free Soluble Forms of the HLA-G Receptors, ILT2 and ILT4. Hum Immunol (2016) 77(9):720–6. doi: 10.1016/j.humimm.2016.01.017 26874236

[107] Desgrandchamps F LeMaoult J Goujon A Riviere A Rivero-Juarez A Djouadou M. Prediction of Non-Muscle-Invasive Bladder Cancer Recurrence by Measurement of Checkpoint HLAG's Receptor ILT2 on Peripheral CD8(+) T Cells. Oncotarget (2018) 9(69):33160–9. doi: 10.18632/oncotarget.26036 PMC6145700 30237859

[108] Rouas-Freiss N LeMaoult J Verine J Tronik-Le Roux D Culine S Hennequin C. Intratumor Heterogeneity of Immune Checkpoints in Primary Renal Cell Cancer: Focus on HLA-G/Ilt2/Ilt4. Oncoimmunology (2017) 6(9):e1342023. doi: 10.1080/2162402X.2017.1342023 28932645 PMC5599087

[109] Zhang X Lin A Han Q Zhang J Chen Q Ye-Y. Intratumor Heterogeneity of HLA-G Expression in Cancer Lesions. Front Immunol (2020) 11:565759. doi: 10.3389/fimmu.2020.565759 33329527 PMC7717930

[110] Zhang S Tao Wang H. Association Between HLA-G 14-Bp Insertion/Deletion Polymorphism and Cancer Risk: A Meta-Analysis. J BUON (2014) 19(2):567–72. 24965423

[111] Crocchiolo R Ringden O Bay J Blaise D Omasic B Mazzi B. Impact of HLA-G Polymorphism on the Outcome of Allogeneic Hematopoietic Stem Cell Transplantation for Metastatic Renal Cell Carcinoma. Bone Marrow Transplant (2018) 53(2):213–8. doi: 10.1038/bmt.2017.243 29131154

[112] Castelli EC Mendes-Junior CT Deghaide NHS de Albuquerque RS Muniz YCN Simões RT. HLA-G Polymorphism and Transitional Cell Carcinoma of the Bladder in a Brazilian Population. Tissue Antigens (2008) 72(2):149–57. doi: 10.1111/j.1399-0039.2008.01091.x 18721275

[113] Gonen-Gross T Achdout H Gazit R Hanna J Mizrahi S Markel G. Complexes of HLA-G Protein on the Cell Surface Are Important for Leukocyte Ig-Like Receptor-1 Function. J Immunol (2003) 171(3):1343–51. doi: 10.4049/jimmunol.171.3.1343 12874224

[114] Ajith A Portik-Dobos V Nguyen-Lefebvre AT Callaway C Horuzsko DD Kapoor R. HLA-G Dimer Targets Granzyme B Pathway to Prolong Human Renal Allograft Survival. FASEB J (2019) 33(4):5220–36. doi: 10.1096/fj.201802017R PMC6436663 30620626

[115] Park JG Na M Kim M Park SH Lee HJ Kim DK. Immune Cell Composition in Normal Human Kidneys. Sci Rep (2020) 10(1):15678. doi: 10.1038/s41598-020-72821-x 32973321 PMC7515917

[116] Balar AV Galsky MD Rosenberg JE Powles T Petrylak DP Bellmunt J. Atezolizumab as First-Line Treatment in Cisplatin-Ineligible Patients With Locally Advanced and Metastatic Urothelial Carcinoma: A Single-Arm, Multicentre, Phase 2 Trial. Lancet (2017) 389(10064):67–76. doi: 10.1016/S0140-6736(16)32455-2 27939400 PMC5568632

[117] Bellmunt J de Wit R Vaughn DJ Fradet Y Lee J Fong L. Pembrolizumab as Second-Line Therapy for Advanced Urothelial Carcinoma. N Engl J Med (2017) 376(11):1015–26. doi: 10.1056/NEJMoa1613683 PMC5635424 28212060

[118] Darvin P Toor SM Sasidharan Nair V Elkord E. Immune Checkpoint Inhibitors: Recent Progress and Potential Biomarkers. Exp Mol Med (2018) 50(12):1–11. doi: 10.1038/s12276-018-0191-1 PMC6292890 30546008

[119] Donini C D'Ambrosio L Grignani G Aglietta M Sangiolo D. Next Generation Immune-Checkpoints for Cancer Therapy. J Thorac Dis (2018) 10(Suppl 13):S1581–601. doi: 10.21037/jtd.2018.02.79 PMC5994499 29951308

[120] Martinez Chanza N Xie W Issa M Dzimitrowicz H Tripathi A Beuselinck B. Safety and Efficacy of Immune Checkpoint Inhibitors in Advanced Urological Cancers With Pre-Existing Autoimmune Disorders: A Retrospective International Multicenter Study. J Immunother Cancer (2020) 8(1):e000538. doi: 10.1136/jitc-2020-000538 32217762 PMC7174076

[121] Tung I Sahu A. Immune Checkpoint Inhibitor in First-Line Treatment of Metastatic Renal Cell Carcinoma: A Review of Current Evidence and Future Directions. Front Oncol (2021) 11:707214. doi: 10.3389/fonc.2021.707214 34527581 PMC8435744

[122] Tronik-Le Roux D Sautreuil M Bentriou M Vérine J Palma MB Daouya M. Comprehensive Landscape of Immune-Checkpoints Uncovered in Clear Cell Renal Cell Carcinoma Reveals New and Emerging Therapeutic Targets. Cancer Immunol Immunother (2020) 69(7):1237–52. doi: 10.1007/s00262-020-02530-x PMC11027645 32166404

[123] Braun DA Bakouny Z Hirsch L Flippot R Van Allen EM Wu CJ. Beyond Conventional Immune-Checkpoint Inhibition - Novel Immunotherapies for Renal Cell Carcinoma. Nat Rev Clin Oncol (2021) 18(4):199–214. doi: 10.1038/s41571-020-00455-z 33437048 PMC8317018

[124] Kshirsagar SK Alam SM Jasti S Hodes H Nauser T Gilliam M. Immunomodulatory Molecules Are Released From the First Trimester and Term Placenta via Exosomes. Placenta (2012) 33(12):982–90. doi: 10.1016/j.placenta.2012.10.005 PMC3534832 23107341

[125] Palma MB Tronik-Le Roux D Amín G Castañeda S Möbbs AM Scarafia MA. HLA-G Gene Editing in Tumor Cell Lines as a Novel Alternative in Cancer Immunotherapy. Sci Rep (2021) 11(1):22158. doi: 10.1038/s41598-021-01572-0 34773056 PMC8589947

[126] Jan CI Huang S Canoll P Bruce JN Lin Y Pan C. Targeting Human Leukocyte Antigen G With Chimeric Antigen Receptors of Natural Killer Cells Convert Immunosuppression to Ablate Solid Tumors. J Immunother Cancer (2021) 9(10):e003050. doi: 10.1136/jitc-2021-003050 34663641 PMC8524382

[127] Benicky J Sanda M Brnakova Kennedy Z Grant OC Woods RJ Zwart A. PD-L1 Glycosylation and Its Impact on Binding to Clinical Antibodies. J Proteome Res (2021) 20(1):485–97. doi: 10.1021/acs.jproteome.0c00521 PMC8158060 33073996

[128] McMaster M Zhou Y Shorter S Kapasi K Geraghty D Lim KH. HLA-G Isoforms Produced by Placental Cytotrophoblasts and Found in Amniotic Fluid Are Due to Unusual Glycosylation. J Immunol (1998) 160(12):5922–8. 9637505

[129] Kuroki K Tsuchiya N Shiroishi M Rasubala L Yamashita Y Matsuta K. Extensive Polymorphisms of LILRB1 (ILT2, LIR1) and Their Association With HLA-DRB1 Shared Epitope Negative Rheumatoid Arthritis. Hum Mol Genet (2005) 14(16):2469–80. doi: 10.1093/hmg/ddi247 16014635

[130] Bainbridge DR Ellis SA Sargent IL. HLA-G Suppresses Proliferation of CD4(+) T-Lymphocytes. J Reprod Immunol (2000) 48(1):17–26. doi: 10.1016/S0165-0378(00)00070-X 10996380

[131] van der Meer A Lukassen HGM van Cranenbroek B Weiss EH Braat DDM van Lierop MJ. Soluble HLA-G Promotes Th1-Type Cytokine Production by Cytokine-Activated Uterine and Peripheral Natural Killer Cells. Mol Hum Reprod (2007) 13(2):123–33. doi: 10.1093/molehr/gal100 17121749

[132] Caumartin J Favier B Daouya M Guillard C Moreau P Carosella ED. Trogocytosis-Based Generation of Suppressive NK Cells. EMBO J (2007) 26(5):1423–33. doi: 10.1038/sj.emboj.7601570 PMC1817622 17318190

[133] Alegre E Rebmann V Lemaoult J Rodriguez C Horn PA Díaz-Lagares A. In Vivo Identification of an HLA-G Complex as Ubiquitinated Protein Circulating in Exosomes. Eur J Immunol (2013) 43(7):1933–9. doi: 10.1002/eji.201343318 23589311

[134] Mittal D Gubin MM Schreiber RD Smyth MJ. New Insights Into Cancer Immunoediting and Its Three Component Phases–Elimination, Equilibrium and Escape. Curr Opin Immunol (2014) 27:16–25. doi: 10.1016/j.coi.2014.01.004 24531241 PMC4388310

[135] Kostlin N Ostermeir A Spring B Schwarz J Marmé A Walter CB. HLA-G Promotes Myeloid-Derived Suppressor Cell Accumulation and Suppressive Activity During Human Pregnancy Through Engagement of the Receptor ILT4. Eur J Immunol (2017) 47(2):374–84. doi: 10.1002/eji.201646564 27859042

[136] Yang S Wei Y Sun R Lu W Lv W Xiao X. Umbilical Cord Blood-Derived Mesenchymal Stromal Cells Promote Myeloid-Derived Suppressor Cell Proliferation by Secreting HLA-G to Reduce Acute Graft-Versus-Host Disease After Hematopoietic Stem Cell Transplantation. Cytotherapy (2020) 22(12):718–33. doi: 10.1016/j.jcyt.2020.07.008 32811747

[137] Chen HX Lin A Shen C Zhen R Chen B Zhang X. Upregulation of Human Leukocyte Antigen-G Expression and Its Clinical Significance in Ductal Breast Cancer. Hum Immunol (2010) 71(9):892–8. doi: 10.1016/j.humimm.2010.06.009 20547193

[138] Pan YQ Ruan Y Peng J Han Q Zhang X Lin A. Diagnostic Significance of Soluble Human Leukocyte Antigen-G for Gastric Cancer. Hum Immunol (2016) 77(4):317–24. doi: 10.1016/j.humimm.2016.01.009 26788811

[139] Dumont C Jacquier A Verine J Noel F Goujon A Wu C. CD8(+)PD-1(-)ILT2(+) T Cells Are an Intratumoral Cytotoxic Population Selectively Inhibited by the Immune-Checkpoint HLA-G. Cancer Immunol Res (2019) 7(10):1619–32. doi: 10.1158/2326-6066.CIR-18-0764 31451484

[140] Spurny C Kailayangiri S Altvater B Jamitzky S Hartmann W Wardelmann E. T Cell Infiltration Into Ewing Sarcomas Is Associated With Local Expression of Immune-Inhibitory HLA-G. Oncotarget (2018) 9(5):6536–49. doi: 10.18632/oncotarget.23815 PMC5814230 29464090

[141] LeMaoult J Krawice-Radanne I Dausset J Carosella ED. HLA-G1-Expressing Antigen-Presenting Cells Induce Immunosuppressive CD4+ T Cells. Proc Natl Acad Sci USA (2004) 101(18):7064–9. doi: 10.1073/pnas.0401922101 PMC406466 15103024

[142] LeMaoult J Caumartin J Daouya M Favier B Le Rond S Gonzalez A. Immune Regulation by Pretenders: Cell-to-Cell Transfers of HLA-G Make Effector T Cells Act as Regulatory Cells. Blood (2007) 109(5):2040–8. doi: 10.1182/blood-2006-05-024547 17077329

[143] Gregori S Tomasoni D Pacciani V Scirpoli M Battaglia M Francesca Magnani C. Differentiation of Type 1 T Regulatory Cells (Tr1) by Tolerogenic DC-10 Requires the IL-10-Dependent ILT4/HLA-G Pathway. Blood (2010) 116(6):935–44. doi: 10.1182/blood-2009-07-234872 20448110

[144] Fournel S Aguerre-Girr M Huc X Lenfant F Alam A Toubert A. Cutting Edge: Soluble HLA-G1 Triggers CD95/CD95 Ligand-Mediated Apoptosis in Activated CD8+ Cells by Interacting With CD8. J Immunol (2000) 164(12):6100–4. doi: 10.4049/jimmunol.164.12.6100 10843658

[145] Rajagopalan S Bryceson YT Kuppusamy SP Geraghty DE van der Meer A Joosten I. Activation of NK Cells by an Endocytosed Receptor for Soluble HLA-G. PloS Biol (2006) 4(1):e9. doi: 10.1371/journal.pbio.0040009 16366734 PMC1318474

[146] van der Meer A Lukassen HGM van Lierop MJC Wijnands F Mosselman S Braat DDM. Membrane-Bound HLA-G Activates Proliferation and Interferon-Gamma Production by Uterine Natural Killer Cells. Mol Hum Reprod (2004) 10(3):189–95. doi: 10.1093/molehr/gah032 14981146

[147] LeMaoult J Zafaranloo K Le Danff C Carosella ED. HLA-G Up-Regulates ILT2, ILT3, ILT4, and KIR2DL4 in Antigen Presenting Cells, NK Cells, and T Cells. FASEB J (2005) 19(6):662–4. doi: 10.1096/fj.04-1617fje 15670976

[148] Perez-Villar JJ Melero I Navarro F Carretero M Bellón T Llano M. The CD94/NKG2-A Inhibitory Receptor Complex Is Involved in Natural Killer Cell-Mediated Recognition of Cells Expressing HLA-G1. J Immunol (1997) 158(12):5736–43. 9190923

[149] Pende D Sivori S Accame L Pareti L Falco M Geraghty D. HLA-G Recognition by Human Natural Killer Cells. Involvement of CD94 Both as Inhibitory and as Activating Receptor Complex. Eur J Immunol (1997) 27(8):1875–80. 10.1002/eji.1830270809 9295021

[150] Xu HH Yan WH Lin A. The Role of HLA-G in Human Papillomavirus Infections and Cervical Carcinogenesis. Front Immunol (2020) 11:1349. doi: 10.3389/fimmu.2020.01349 32670296 PMC7330167

[151] Heidegger I Borena W Pichler R. The Role of Human Papilloma Virus in Urological Malignancies. Anticancer Res (2015) 35(5):2513–9. 25964524

